# Location of sources in reaction-diffusion equations using support vector machines

**DOI:** 10.1371/journal.pone.0225593

**Published:** 2019-12-05

**Authors:** Venecia Chávez-Medina, José A. González, Francisco S. Guzmán

**Affiliations:** Laboratorio de Inteligencia Artificial y Supercómputo, Instituto de Física y Matemáticas, Universidad Michoacana de San Nicolás de Hidalgo. Edificio C-3, Cd. Universitaria, 58040 Morelia, Michoacán, México; Instituto Nacional de Medicina Genomica, MEXICO

## Abstract

The reaction-diffusion equation serves to model systems in the diffusion regime with sources. Specific applications include diffusion processes in chemical reactions, as well as the propagation of species, diseases, and populations in general. In some of these applications the location of an outbreak, for instance, the source point of a disease or the nest of a vector spreading a virus is important. Also important are the environmental parameters of the domain where the process diffuses, namely the space-dependent diffusion coefficient and the proliferation parameter of the process. Determining both, the location of a source and the environmental parameters, define an inverse problem that in turn, involves a partial differential equation. In this paper we classify the values of these parameters using Support Vector Machines (SVM) trained with numerical solutions of the reaction-diffusion problem. Our set up has accuracy of classifying the outbreak location above 90% and 77% of classifying both, the location and the environmental parameters. The approach presented in our analysis can be directly implemented by measuring the population under study at specific locations in the spatial domain as function of time.

## 1 Introduction

The reaction-diffusion (RD) equation is applied to model various dynamical systems involving populations. For instance in mathematical biology, the RD equation is applied, in the frame of biological invasion, used to model the propagation of dengue via the Aedes aegypti vector at urban spatial scale [1]. More recently, a free boundary approach is used to model the behavior Aedes aegypti mosquito populations, based on reaction diffusion advection processes to determine the population spreading and vanishing regimes [2]. Also the propagation and proliferation of other types of viruses like the West Nile virus [3, 4] or the feline immunodeficiency virus [5], is studied using RD models. At metropolitan scale, the RD system is used to model the population dynamics of the tiger mosquito and has potential at the scale of a whole country [6]. Generalization to more than one population is constructed by coupling two or more RD equations, and is useful to explain the spread of seeds and animals. For instance, RD allows the description of cross-diffusion in multiple species systems with nonlocal interaction [7], and modeling biological invasion [8, 9]. In fact, the particular case of cross-diffusion has a wide variety of applications in mathematical biology [10]. The RD is also being used to model the response of ecosystems to climate change, for instance through the rainfall decline [11].

With different degrees of complexity and additional conditions, the temporal and spatial evolution of a population *u* is assumed to be described by the RD equation

$$\begin{array}{c} \frac{\partial u}{\partial t}=\nabla \cdot (D(x)\nabla u)+s\left(u\right). \end{array}$$ (1)

The evolution of the population *u* is described by the solution of an initial value problem for given initial conditions *u* (**x**, 0) = *u*_0_ (**x**) evolving in a domain $A\in R^{2}$. The first term of the right hand side is the diffusion operator with a space-dependent diffusion coefficient *D*(**x**), and *s*(*u*) is a source term usually named proliferation term when *u* is interpreted as the density of a population. The space dependency of the diffusion *D* and *s* is useful in various scenarios. For example, the spread of a species of seeds or animals, depends on environmental variables that influence the diffusion and proliferation effects (see e.g. [12, 13]).

Of course, different initial conditions, as well as different diffusion and proliferation functions, give rise to different solutions *u*(**x**, *t*). The solution of the *direct problem* consists in the evolution of an initial profile *u*_0_(**x**), for instance a fluctuation representing the outbreak of a disease or a vector, with a specific diffusion coefficient function *D*(**x**) representing the spatial conditions for the propagation of the population, and the proliferation function *s*(*u*) associated to a population growth model. Solving the problem consists in calculating the value of *u* at a given set of particular points in the domain called detectors, and predict the population *u* in a spatial neighborhood of such detectors as function of time *t*. For the particular problem of the RD equation, this can be achieved by numerically solving the initial value problem (IVP) in (1).

The direct problem is of interest in itself when *D* and *s* are known for specific initial data. It happens though that locating the position of an outbreak’s origin, the starting point of a plague, of an anomaly in seed spread based on measurements, are also important situations, with the additional difficulty of not knowing the initial conditions that provoked the observations and possibly including ignorance on the parameters of *D* and *s* of a specific disease and population. These scenarios correspond to *inverse problems* focused on the reconstruction of the initial conditions and environmental variables. Inverse problems related to determine parameters and initial conditions associated to partial differential equations using Artificial Neural Networks happen in different areas, for instance in fluid dynamics [14, 15]. The aim of this paper is to present a strategy to solve this inverse problem by combining the numerical solution of the IVP (1) and the use of SVMs to classify and bound the initial conditions and environmental variables.

The paper is organized as follows. In Section 2 we present the details for the solution of the initial value problem of the RD equation and the machine learning methods. In Section 3 we present the results for the location of an outbreak origin and the environmental parameters. Finally in Section 4 we draw some conclusions.

## 2 Direct and inverse problems

### 2.1 General description of the inverse and direct problems

We will study the solution of two inverse problems. The first one consists in the location of a fluctuation of the population *u*, that could represent the origin of an outbreak. One counts with measurements in time of *u* at various points of the domain **x**_*det*_ ∈ *A* called detectors and the knowledge that the process is governed by Eq (1) for given *D*(**x**) and *s*(*u*(**x**)). Should the problem be linear, the location of the outbreak could make use of a triangulation, nevertheless the equation and the possible space dependency of the diffusion, complicate the implementation of a straightforward triangulation.

The second inverse problem we analyze consists in the use of the time series measured by the detectors, and the objective is to determine not only the location of the source, but also the parameters of *D*(**x**) and *s*(*u*(**x**)).

In this paper we use the SVM to solve these two problems using the same strategy. The approach to their solution consists in a classification scheme. This means that a training set is constructed with location and environmental parameters within specific ranges known as classes. The method used to solve these inverse problems is based on SVM method that requires training. Training is based on the solution of the direct problem in the following systematic way.

#### Training the SVM for the location of the outbreak

We solve the direct problem for a set of initial conditions with a fluctuation of the population *u* centered at known positions $x_{src}^{i,j}$ where *i*, *j* are tags for the location of the fluctuation on a two dimensional domain *A*. Then measure the value of the solution *u* at detector locations $x_{det}^{k,l}$ as a function of time $u(x_{det}^{k,l},t)$, where *k*, *l* are now tags for the position of the detectors on *A*. The set of time-series measured by detectors $u(x_{det}^{k,l},t)$ corresponding to the different source locations $x_{src}^{i,j}$ are used to train the SVM and fix its internal parameters.

#### Training for the second inverse problem

For the additional determination of *D* and *s* we construct a training set of runs for different values of *D* and *s* that produce time series again measured at detector locations $x_{det}^{k,l}$. These time-series are used to train the SVM as before. The difference is that in this case the locations of initial fluctuations $x_{src}^{i,j}$ are distributed in classes, and the values of *D* and *s* are distributed in classes as well.

After the training process, a set of measurements known as prediction set, constructed with location and environmental parameters different from those used to construct the training set, are presented to the SVM which will determine the class they should belong to, that is, a class associated with the position of the source, diffusion coefficient, and proliferation parameter. The classification can be correct or incorrect, and the amount of correct classifications determines the accuracy of the method for a prediction set.

### 2.2 The direct problem

The direct problem we solve is an IVP involving (1) on the spatial domain $B\subseteq R^{2}$, for *t* ∈ [0, *t*_*max*_], provided initial conditions *u*_0_(**x**) and boundary conditions at ∂*B*. For our analysis we set the spatial domain to *B* = {(*x*, *y*) ∈ [−2, 2] × [−2, 2]} and impose the boundary conditions $\hat{n}\cdot \nabla u=0$ at the boundary ∂*B*, that in theory allow the numerical boundaries to be transparent. Even if these boundary conditions are imposed to guarantee the uniqueness of the solution, we improve the transparency of the boundaries for our problem, by defining the subset *A* = {(*x*, *y*) ∈ [−1, 1] × [−1, 1]} ⊂ *B* as the domain of analysis. This means that we will use initial data consisting of localized fluctuations of *u*_0_(**x**) centered at positions $x_{src}^{i,j}$ and detectors $x_{det}^{k,l}$ located also only within *A*.

The value of *t*_*max*_ is such that the diffusion of the initial fluctuation in the initial conditions *u*_0_(**x**), reaches the whole domain *A*, and at the same time the possible spurius noise reflected from ∂*B* does not reach the boundary ∂*A*. In this manner we prevent the values of *u* measured at the detectors from being influenced by boundary effects because ∂*B* is causally disconnected from the domain of interest *A*. For the estimates of time travel of the signals across the domain, we consider the velocity of the signal to be the Fischer-Kolmogorov velocity, suitable for the reaction diffusion equation [16, 17].

We then solve this IVP numerically using a finite differences approximation of (1), with second order accurate stencils, defined on a discrete domain version of *B*, with constant and uniform resolutions Δ*x* = Δ*y* = 0.01. The evolution in time is solved out using the method of lines with a third order accurate TVD Runge-Kutta integrator, and time resolution Δ*t* [18]. In order to keep the stability of the evolution method we choose the time step such that $\Delta t\leq \min(\frac{\Delta x^{2}}{2D})$.

#### Diffusion

In order to construct a more realistic scenario, with a region with a larger diffusion coefficient than in the rest of the domain, that could for instance represent a zone with favorable conditions for reproduction or diffusion, like humidity of a river that favors the diffusion of some bacteria or the proliferation of mosquitoes [19], also thought of as corridors [20], we define a model for the diffusion function to be

$$\begin{array}{c} D\left(x\right)=D_{1}+D_{2}e^{-(\frac{x^{2}+y^{2}-2xy}{2\sigma^{2}})}. \end{array}$$ (2)

where **x** = (*x*, *y*). We set the width of the Gaussian corridor to *σ* = 0.08 and the reason for this value is that we consider the accuracy in the location of an outbreak is interesting within a scale size of the corridor. Now, since we will launch a set of simulations with initial fluctuations centered at positions $x_{src}^{i,j}$ separated by a distance of order ∼0.2, our value of *σ* is appropriate. In our analysis the value of *σ* is kept fixed in all of our runs, whereas there are two free parameters *D*_1_ and *D*_2_ that could depend on the environmental conditions or characteristics of the population.

#### Proliferation

For the proliferation we use the logistic population model *s*(*u*) = *ρu*(1 − *u*), where *ρ* is the normalized birth-death coefficient. We also want this coefficient to depend on the environmental conditions and use a function that enhances proliferation in the region with more diffusion, for which we use a similar Gaussian strip as before, across the numerical domain given by

$$\begin{array}{c} \rho \left(x\right)=0.5+\rho_{0}e^{-(\frac{x^{2}+y^{2}-2xy}{2\sigma^{2}})}. \end{array}$$ (3)

with the same value of *σ* as for the diffusion. In our analysis we assume *σ* is fixed, whereas *ρ*_0_ is a parameter we vary in our analysis, that indicates how much the population *u* proliferates.

### 2.3 The training set

The training set is prepared with the solution of the direct problem for an educated set of values of the parameters *D*_1_ or *D*_2_ in the diffusion model (2), *ρ*_0_ for the proliferation model (3) and the two coordinates of the center of the outbreak location $x_{src}^{i,j}$.

#### Initial conditions

**Fig 1:**
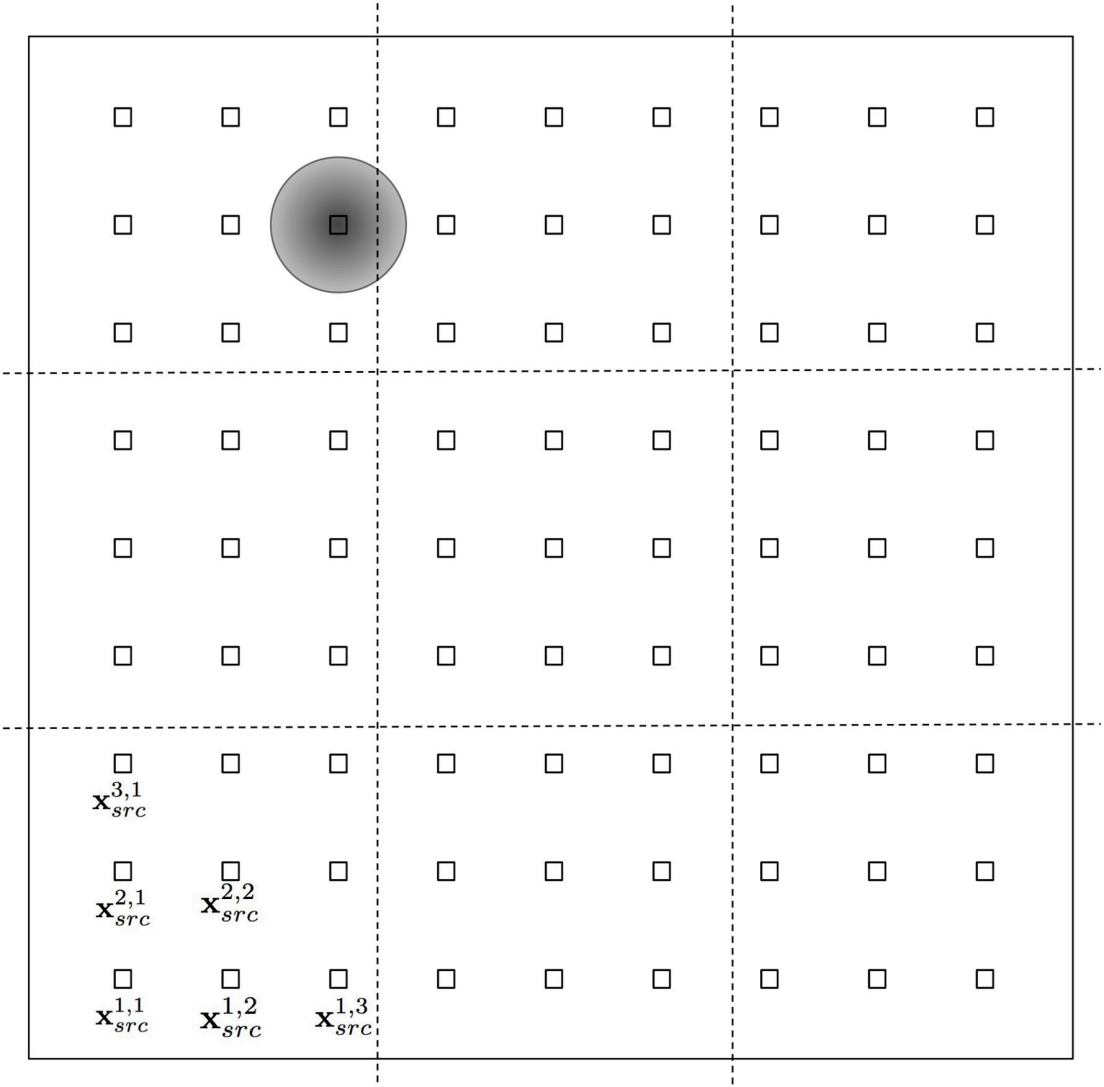
Numerical domain *A* = [−1, 1] × [−1, 1] and the position of the outbreak sources with coordinates $x_{src}^{i,j}$ indicated with squares. For the sake of illustration we only label a few of them in the bottom-left corner. The shaded circle indicates the location of the outbreak location for the fluctuation case centered at $x_{src}^{3,8}$.

Since we want to simulate an outbreak type of process we define initial conditions with a prominence at a given location $x_{src}^{i,j}$. For this we decided to use a Gaussian pulse

$$\begin{array}{c} u_{0}\left(x\right)=u_{0}\left(x_{src}^{i,j}\right)=\text{exp}\left(-{\left(x-x_{src}^{i,j}\right)}^{2}/\sigma_{src}^{2}\right) \end{array}$$ (4)

where $x_{src}^{i,j}$ is the center of the outbreak location. Specifically, the initial conditions are characterized only by the location of the outbreak parametrized by the two coordinates of $x_{src}^{i,j}$. We define 9 values for each of the two coordinates, so that the center of the Gaussian is located at different points uniformly distributed across the domain *A*, so that for *i*, *j* = 1, …, 9 with

$$\begin{array}{c} x_{src}^{i,j}=(-1+(i+\frac{1}{2}),-1+(j+\frac{1}{2}))\Delta s \end{array}$$ (5)

where Δ*s* = 2/10 is the length of the domain along the *x* or *y* directions divided by 10. In order to avoid a considerable overlap in the initial fluctuations launched from neighboring outbreak center positions $x_{src}^{i,j}$ we use *σ*_*src*_ = 0.04. In Fig 1 we present the domain *A* and the location of the 81 positions $x_{src}^{i,j}$ where the initial fluctuation is centered. The gray circle illustrates the position of the initial Gaussian population profile for the particular case $x_{src}^{3,8}$.

#### Classes

Nine squared equal size classes are defined for the location of the sources, that correspond to different non overlapping parcels of the domain that cover the whole area of *A*. These classes are delimited by the dashed lines in Fig 1. For example, sources at $x_{src}^{1,3}$ and $x_{src}^{3,1}$ belong to the bottom left class, whereas $x_{src}^{4,4}$ belongs to the class in the center of the domain.

#### Environmental and population parameters

**Fig 2:**
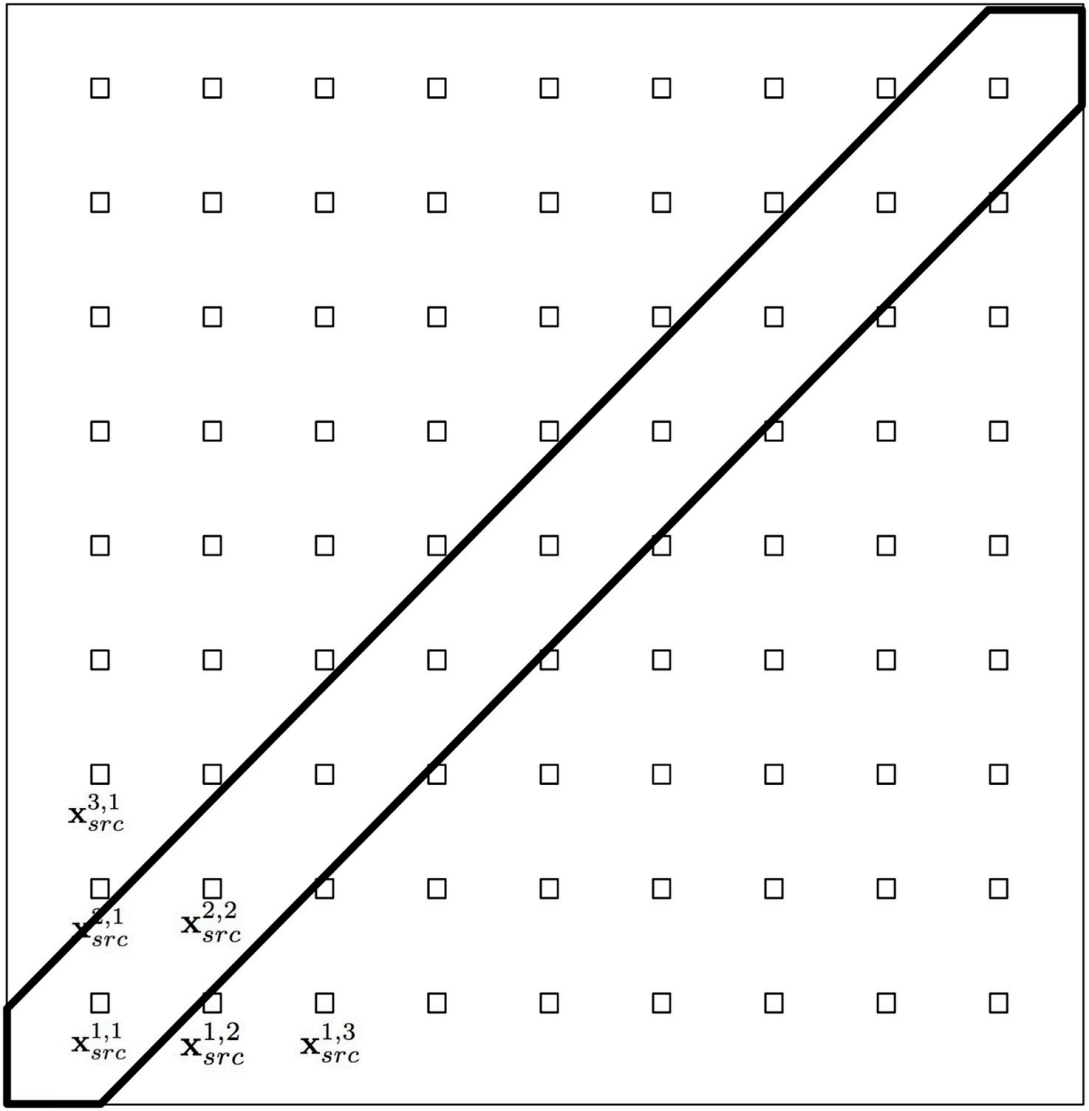
Numerical domain *A* = [−1, 1] × [−1, 1] with a diagonal strip where the diffusion coefficient is bigger according to Eq (2).

For the diffusion parameters, notice that in (2) *D*_1_ is a background value whereas *D*_2_ is the amplitude of the Gaussian prominence along a diagonal of the domain. We illustrate the distribution of the diffusion function in Fig 2.

We fix the value of *D*_1_ and set the value of *D*_2_ free. For this we define the fixed value *D*_1_ = 0.1 and let *D*_2_ ∈ [0, 1.9] so that the maximum ratio in different regions of the domain is 20, which is a wide range. Specifically we use the values $D_{2}^{k}=k\Delta D$ with *k* = 0, …, 8 and Δ*D* = 1.9/8. The classes for the value of *D*_2_ are defined as three equal sized segments of the domain *D*_2_ ∈ [0, 1.9].

We also define nine values for the proliferation parameter *ρ*_0_ following the expression

$$\begin{array}{ccc} \rho_{0}^{l} & = & \rho_{min}+l\Delta \rho \;\;\;\;\;l=0,1,\ldots ,8\\ \Delta \rho & = & \frac{\rho_{max}-\rho_{min}}{8} \end{array}$$ (6)

where *ρ*_*min*_ = −0.5 and *ρ*_*max*_ = 0.5, which includes positive and negative population grow rate. Like in the previous cases we define three classes for the value of equal size segments of the domain *ρ*_0_ ∈ [−0.5, 0.5].

In total we have defined 81 classes for the location, diffusion and proliferation parameter space.

#### Example of a solution to the direct problem

**Fig 3:**
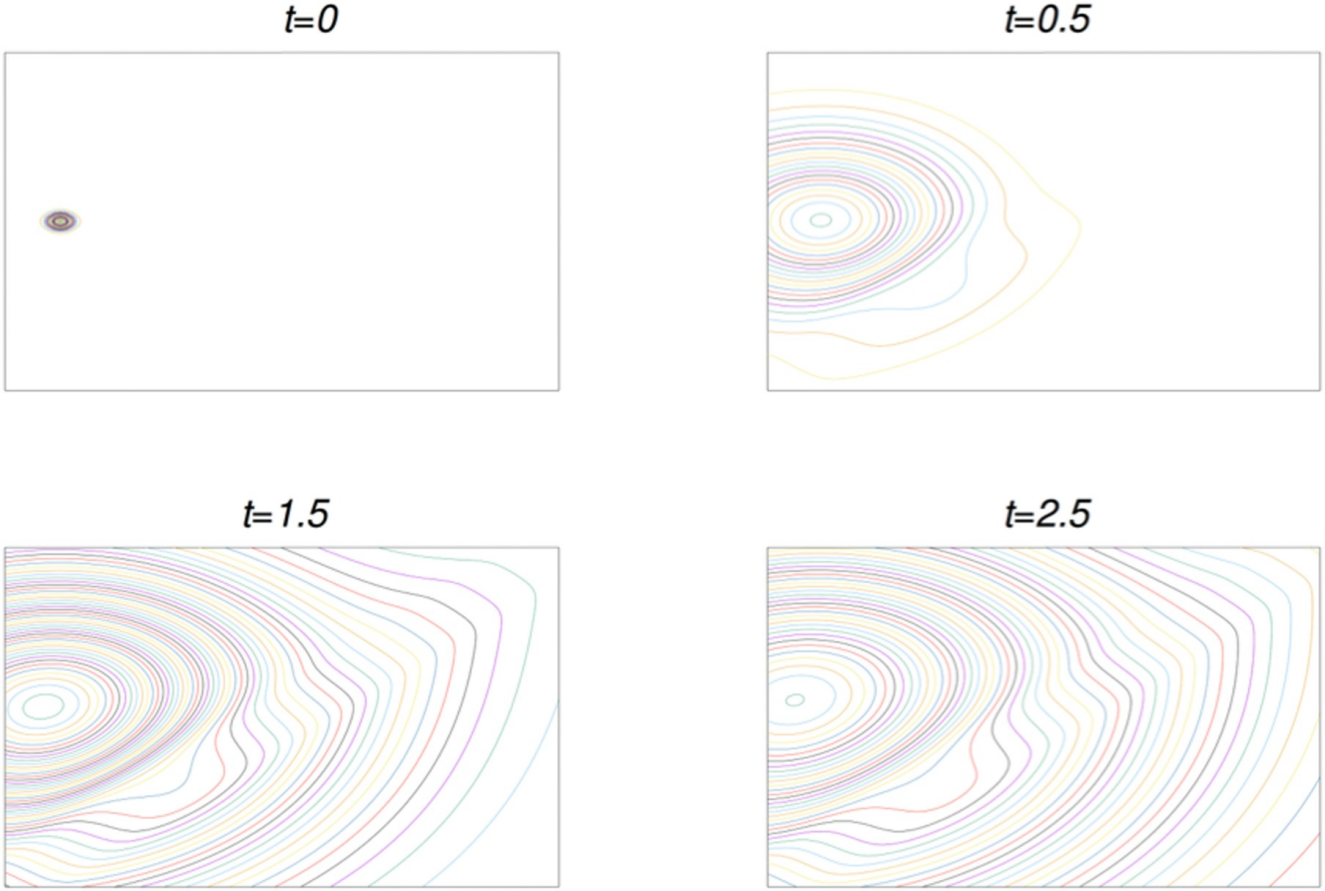
Snapshot for the isocontours of *u* at different times, for the case *D*_2_ = 0.95, *ρ*_0_ = −0.25. In this example the outbreak is centered at $x_{src}^{5,1}$. The domain of the plots is *A*.

In Fig 3 we show the iso-contours for the solution of the direct problem for the outbreak centered at position $x_{src}^{1,5}$, with environmental parameters *D*_2_ = 0.95 and *ρ*_0_ = −0.25. At initial time *u* shows the profile in (4), which is illustrated with the first snapshot. Later on *u* propagates across the domain. The space-dependent diffusion, higher along the diagonal according to Eq (2) and Fig 2, allows a different velocity of propagation. Notice that the distortion of the lines is pronounced along the diagonal where the diffusion is higher from *t* = 0.5 and on. Notice that by the time of the last snapshot, the signals have reached all the detectors. This is the type of problem we solve for all the combinations of source locations, values of *D*_2_ and *ρ*_0_ described previously.

However the solution itself is not the information collected in measurements of a real case scenario. The information will consist in the value of *u* at certain given points of the domain as a function of time measured by detectors.

#### Detectors

**Fig 4:**
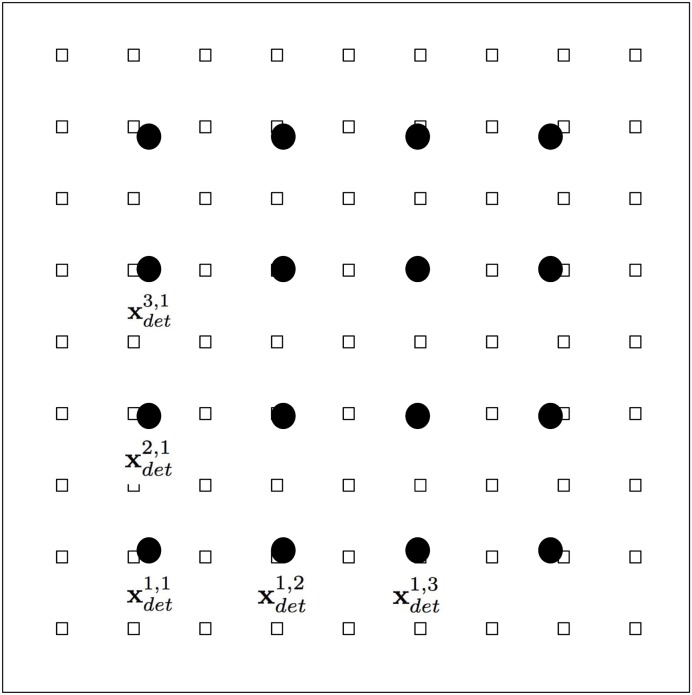
Location of the 81 outbreak positions indicated with small squares and the 16 detectors as filled circles. For illustration, only a few of the detector locations are labeled.

We locate detectors that record the value of *u* at fixed specific locations. In an applied example, these could be the location of an infectious vector counter for instance. In our numerical example, we locate 16 of these detectors in the following positions $x_{det}^{k,l}=\left(x_{k},y_{l}\right)$ where *x*_*k*_, *y*_*l*_ = −0.6, −0.2, 0.2, 0.6. In Fig 4 we show the location of the outbreak points and the location of the detectors $x_{det}^{k,l}$ with filled circles. These detectors measure the function $u(x_{det}^{k,l},t)$ in the time domain. The specific data collected by these detectors is given within the time domain *t* ∈ [0, 2.5] and registered every 0.01 units of time. In this way, each detector collects 250 values of *u*.

**Fig 5:**
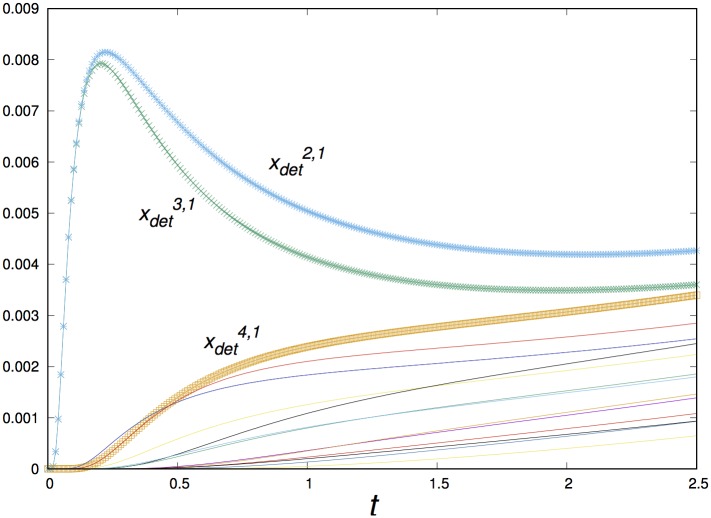
Time series of the variable *u* measured at the 16 detectors $u(x_{det}^{k,l},t)$ measured by the 16 detectors for *D*_2_ = 0.95, *ρ*_0_ = −0.25, and outbreak centered at $x_{src}^{5,1}$. As described in the text, each detector captures 250 values of *u* in the time domain.

An example of the time series measured by the detectors is shown in Fig 5. The initial and environmental conditions are the same used to construct Fig 3. We indicate labels for the measurements corresponding to the detectors that are close to the outbreak location. It is helpful to compare the source center at $x_{src}^{1,5}$ in Fig 1 and the location of the closest detectors to this point in Fig 4. The signal arrives later to the other detectors, with different time dependence and amplitudes.

Finally, with these specifications, the training set consists of the results generated by all the combinations of the parameters of position, diffusion and proliferation defined above. Each of them has 9 values, which means that we solve the direct problem 9^4^ times, and produce the time series of 250 data values measured by each of the 16 detectors. These 16 × 9^4^ time series constitute the set used to train the SVM in the most general case, for the second of the inverse problems. For the first inverse problem, the training set is constructed with the same settings as for the general case, except that *D*_2_ and *ρ*_0_ are set fixed.

### 2.4 The SVM classification method

Among the various machine learning methods, we account with implementations of artificial neural networks (ANNs) as regressors and classifiers, so as convolutional neural networks (CNNs). The use of ANNs has the advantage of easy implementation and high adaptability to non-linear problems. Nevertheless, the computational time required to train the network grows with the number of inputs, in our case, the data set in the 16 time series measured by the detectors; it also grows with the number of neurons in the hidden layers; finally it also increases with the number of classes. The number of inputs and classes used in our analysis is prohibitive for ANNs, both as classifiers or regressors. A second option are the CNNs, which are known for their efficiency at classifying images. In this case we deal with time-series instead of images, which is the reason we prefer to use Support Vector Machines.

The specific SVM method used in our analysis, consists in constructing the optimal decision function by obtaining the solution of an optimization problem subject to constraints with a radial basis function kernel with two free parameters. In order to select the best possible values for these parameters, we perform a parameter search known as *cross-validation*. Basically, the cross-validation separates the training set into a fixed number of subsets and, sequentially, each one of those subsets is used to test the accuracy of the decision function obtained using the rest of the subsets. For a detailed explanation of the methods the reader can consult [21, 22] and for a specific setup of our implementation in a similar analysis [23]. Instead of implementing our own version of the support vector machine, we use the library libSVM [22].

## 3 Results

We aim to classification of two inverse problems, the one focused on the location of the outbreak position only, and the second one intended for the classification of the four parameters.

The key performance measure used in our analysis is the percentage of correctly classified cases within the prediction set. The accuracy is the average of accuracy results obtained using various randomly generated prediction sets, with the aim of minimizing fluctuations.

### 3.1 Location of the source only

#### Training accuracy

The SVM was trained with all the time series registered by detectors considering the 81 source locations $x_{src}^{i,j}$ according to Eq (5). The accuracy in classification of the SVM at training was above 95%. The SVM with this training is the one used to estimate the accuracy in prediction.

#### Prediction sets

In this case the prediction set consists of 225 simulations with randomly generated coordinate position of the source $x_{src}^{pred}$ and fixed values of diffusion and proliferation parameters *D*_1_, *ρ*_0_. We make sure the coordinates do not coincide with any of the positions $x_{src}^{pred}\neq x_{src}^{i,j}$ used for training, which is a modelling assumption that avoids the trivial cases. When the SVM is used to classify the outbreak location only, the accuracy of the method at prediction is 90.0%.

### 3.2 Location of the source plus environmental parameters

#### Training accuracy

In this case the SVM was trained with the time series of the detectors considering the 81 source locations, the 9 values of *D*_2_ and the 9 values of *ρ*_0_ defined before. In total 81^2^ simulations for 9^2^ classes. The accuracy in classification at training was above 80%.

#### Prediction

In this case we prepared 5 different sets with 1000 runs each, using random values of location coordinates, *D*_2_ and *ρ*_0_, within the domain values used so far, but unknown to the SVM. The average accuracy among the prediction sets in the four parameter case is of 77.72%.

## 4 Conclusions and final comments

We have presented the effectiveness of an SVM to classify the location of an outbreak, and parameters of diffusion and proliferation of a process ruled by the RD equation in a domain. These parameters are constructed out of measurements obtained at given detectors in the domain of interest.

When a set of parameters is correctly classified it means that such parameter values lie within the range associated with the class selected by the SVM. For example, the domain *A* = [−1, 1] × [−1, 1] is covered by nine classes corresponding to squares of sides 2/3. When the SVM correctly classifies the location of the outbreak, it means that the possible location will be associated to the center of the class ±1/3 along each direction. The same argument is applied for the proliferation and diffusion parameters.

Improvement in accuracy needs classes of smaller sizes. For a fixed domain like *A*, it means the use of a bigger set of simulations for training, which is affordable if the problem consists only in the location of the outbreak. For example, it is easy to cover the domain with 6 × 6 classes instead of 3 × 3 to reduce the uncertainty from ±1/3 to ±1/6 in the location of the source. The price that has to be paid is to increase the number of elements in the training set by a factor of 2^2^. Nevertheless when the parameter space is four dimensional the number of elements in the training set has to be increased to 2^4^ which is unpractical.

**Fig 6:**
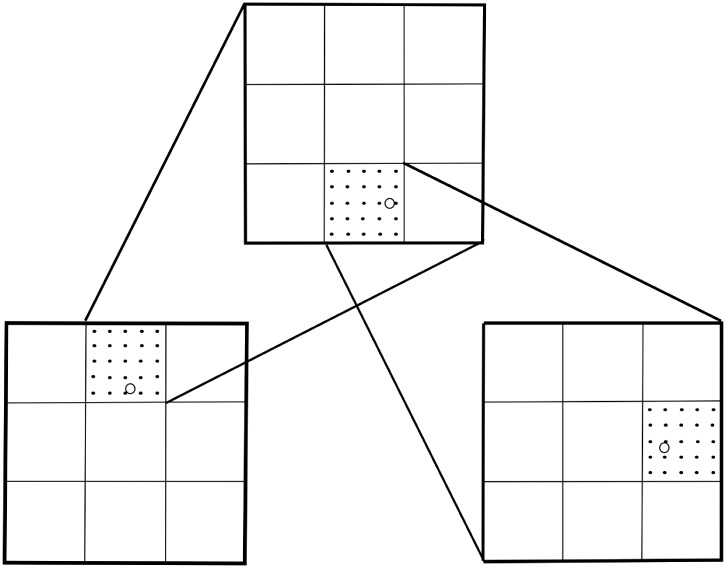
This figure illustrates how the method can be used on refined subdomains in order to reduce the uncertainty of the parameters in each class.

Instead, to decrease the uncertainty in the values of parameters, we can use a mesh refinement of the parameter space, which implies a reduction in size for a specific class, and consequently, the reduction of uncertainty. For example, in Fig 6 we illustrate with an example in two dimensions the case in which the SVM predicts that the source of an outbreak lies in the up-center class (see left-bottom square). A refinement of this domain is done and a new training within this subdomain determines that the source of the outbreaks will be located at the bottom-center class of that subdomain (see center-up square). In a further refinement, the SVM indicates that the source is located in the center-right class (see right-bottom square). With this process we have decreased the uncertainty in the prediction by a factor of 1/9. This procedure has been successfully implemented in other classification problems such as [24, 25]. In fact, this is a strategy to tackle inverse problems associated with initial value problems ruled by partial differential equations with a potential variety of applications.

Finally, considering a real scenario problem, like the spread of a vector that transmits a disease, the domain could be not as regular as the one used here, and the detectors may not be located in such a regular grid type of distribution. Nevertheless, the advantage of the method presented here is that it can be easily generalized to general shaped domains and irregular distributions of detectors. Two immediate applications of our method can be the propagation of mosquitoes at country and city scale including corridor components [6] and modeling the evolution of environmental parameters in ecological systems due to the impact of climate change [11].

With respect to the computing power required for this analysis, all the runs, those involving the numerical solution of the RD problem, so as the runs with SVM were carried out in regular computers equipped with 2.50GHz Xeon processors without any special requirements.
